# The superior beneficial effects of exercise training versus hormone replacement therapy on skeletal muscle of ovariectomized rats

**DOI:** 10.1038/s41598-022-12739-8

**Published:** 2022-05-24

**Authors:** Sara Barros Silva, Kinulpe Honorato-Sampaio, Sabrina Paula Costa, Talita Emanuela Domingues, Timilly Mayra Martins da Cruz, Cíntia Maria Rodrigues, Karine Beatriz Costa, Jousielle Márcia dos Santos, Vanessa Kelly da Silva Lage, Thais Peixoto Gaiad, Ana Paula Santos, Marco Fabrício Dias-Peixoto, Cândido Celso Coimbra, Adelina Martha dos Reis, Raphael Escorsim Szawka, Pedro Henrique Scheidt Figueiredo, Henrique Silveira Costa, Murilo Xavier Oliveira, Vanessa Amaral Mendonça, Ana Cristina Rodrigues Lacerda

**Affiliations:** 1 Programa de Pós-Graduação em Reabilitação e Desempenho Funcional (PPGReab), Universidade Federal dos Vales do Jequitinhonha e Mucuri (UFVJM), Campus JK-Highway MGT-367-Km 583, N°. 5000-Alto da Jacuba, Diamantina, 39100-000 Brazil; 2 Centro Integrado de Pós-Graduação e Pesquisa em Saúde (CIPq-Saúde), Universidade Federal dos Vales do Jequitinhonha e Mucuri (UFVJM), Diamantina, Brazil; 3 Programa Multicêntrico de Pós-graduação Multicêntrico em Ciências Fisiológicas (PPGMCF), Sociedade Brasileira de Fisiologia (SBFis), Diamantina, Brazil; 4 Programa de Pós-Graduação em Ciências da Saúde (PPGCS), Universidade Federal dos Vales do Jequitinhonha e Mucuri (UFVJM), Diamantina, Brazil; 5 Faculdade de Medicina, Universidade Federal dos Vales do Jequitinhonha e Mucuri (UFVJM), Diamantina, Minas Gerais Brazil; 6 Faculdade de Ciências Biológicas e da Saúde, Universidade Federal dos Vales do Jequitinhonha e Mucuri (UFVJM), Diamantina, Minas Gerais Brazil; 7 Instituto de Ciências Biológicas, Universidade Federal de Minas Gerais (UFMG), Belo Horizonte, Minas Gerais Brazil

**Keywords:** Physiology, Ageing

## Abstract

Previous studies have highlighted the positive effects of Estradiol (E2) replacement therapy and physical exercise on skeletal muscle during menopause. However, the comparison effects of exercise training (ET) and estradiol replacement therapy during menopause on skeletal muscle have not been investigated to date. This study aimed to compare the effects of endurance exercise training *versus* E2 replacement therapy on mitochondrial density, redox status, and inflammatory biomarkers in the skeletal muscle of ovariectomized rats. Thirty female *Wistar* rats (12-week-old) were randomly assigned into three groups: Untrained ovariectomized rats (UN-OVX, n = 10); untrained ovariectomized rats treated with estradiol replacement therapy (E2-OVX); and, trained ovariectomized rats (TR-OVX). After ovariectomy, the E2-OVX rats were treated subcutaneously with E2 (implanted Silastic® capsule containing 360 μg of 17β-estradiol/mL) while the TR-OVX group performed an exercise training protocol (50–70% of maximal running speed on a treadmill, 60 min/day, 5 days/week for 8 weeks). After euthanasia, the soleus muscle was processed for histological and biochemical evaluations. Only exercise prevented the reduction of maximal oxygen consumption and increased mechanical efficiency (ME). While mitochondrial muscle density, total antioxidant capacity (FRAP), catalase (CAT) activity, and interleukin 10 levels were higher in TR-OVX, only OVX-E2 presented higher CAT activity and lower interleukin 6 levels. Endurance exercise training compared with E2 replacement therapy maintains the aerobic capacity improving the ME of OVX rats. In addition, only endurance exercise training raises the skeletal muscle mitochondrial content and tends to balance the redox and inflammatory status in the skeletal muscle of OVX rats.

## Introduction

The postmenopausal period is associated with several metabolic and musculoskeletal chronic disorders, such as osteoporosis, tendinopathies, and arthritis^1,2^. Thus, maintaining skeletal muscle integrity during menopause plays a central role in preventing musculoskeletal disorders^1^. The mechanisms through which ovarian hormone deficiency negatively affects the muscle integrity during the postmenopausal period are multifactorial, involving a pro-inflammatory profile and mitochondrial dysfunction^1,2^. Studies using ovariectomized (OVX) rats, the most common experimental menopause model^3^, have reported that the skeletal muscle from OVX rats presents a reduction in mitochondrial biogenesis and an increase in oxidative stress^4,5^. In addition, ovariectomy alters the intramuscular levels of several cytokines. Ovariectomy enhances tumor necrosis factor-alpha (TNF-α) expression and interleukin 6 (IL-6) levels, pro-inflammatory cytokines associated with muscle disfunction^6–9^, and reduces interleukin 10 (IL-10) levels, an anti-inflammatory^8,9^. Additionally, chronic stimulation of pro-inflammatory cytokines, e.g., TNF-α, impairs mitochondrial function and biogenesis, promoting a vicious pro-inflammatory cycle^10,11^.

Many studies have highlighted the positive effects of estrogen replacement therapy during menopause^4,5^. Moreover, a growing body of evidence has also proven several beneficial effects of physical exercise therapy in counteracting the deleterious effects of menopause in the skeletal and cardiac muscles. In previous studies, we and others found that exercise training improved the cardiovascular and cardiac function of OVX rats^5–7^. However, comparison effects of endurance exercise training versus estradiol (E2) replacement therapy after menopause on skeletal muscle mitochondrial profile and redox/inflammatory status have not been investigated to date.

Given that endurance exercise training stimulates mitochondrial biogenesis and improves redox and inflammatory status in the skeletal muscle under physiological and pathological conditions^12–14^, we hypothesized that endurance exercise training would have similar or higher benefits compared to E2 replacement therapy to the skeletal muscle mitochondrial content and the redox and inflammatory status in OVX rats. Thus, we aimed to investigate the effects of endurance exercise training versus E2 replacement therapy on muscle mitochondrial density and redox/inflammatory status in OVX rats.

## Methods

### Animals

12-week-old female *Wistar* rats (n = 30, body mass = 216 ± 2.54 g) were provided by the animal facility of the Universidade Federal de Minas Gerais, Brazil. The rats' age in this study was determined according to previous studies^4,15,16^ and based on the premise that at 12 weeks of age, Wistar female rats are sexually mature^16^.

The animals were maintained in a temperature-controlled room (22 °C), in a 12 h dark: light cycle, and had free access to standard chow (Nuvilab Nutrients LTDA, Colombo, PR, Brazil) and water. All groups received the same diet (free from phytoestrogen) throughout the experimental period. All rats were treated similarly in terms of daily manipulation. All surgical procedures and protocols used were approved by the Animal Use Ethics Committee of the Universidade Federal dos Vales do Jequitinhonha e Mucuri (protocol nº015/2019) and conducted in accordance with the National Institute of Health (NIH) Guide for the Care and Use of Laboratory Animals.

### Experimental design and sampling

The rats were randomly assigned into three groups: (1) Untrained ovariectomized rats (UN-OVX, n = 10), (2) Ovariectomized rats treated with Estradiol replacement (E2-OVX, n = 10), and (3) Trained ovariectomized rats (TR-OVX, n = 10). Of note, all animals were ovariectomized and received a subcutaneously implanted Silastic® capsule containing vehicle (UN-OVX and TR-OVX groups) or 17β-estradiol (E2-OVX group). The UN-OVX and E2-OVX groups were similarly handled and placed near the treadmill during the training sessions of the TR-OVX group to match the same environmental exposure conditions. Two weeks after ovariectomy, TR-OVX rats were submitted to an endurance exercise training protocol for eight weeks. All animals were familiarized with the maximal effort treadmill test and exercise protocol.

The rats were euthanized by decapitation. Both right and left soleus muscles were harvested, washed in ice-cold PBS (0.15 M, pH 7.34), frozen in liquid nitrogen, and stored at – 80 °C. The left soleus muscles were processed for oxidative stress evaluation and the right ones for inflammatory analyses. In addition, posterior mid-belly fragments of the right soleus from three animals per group were dissected and chemically fixed for mitochondrial density assessment by transmission electron microscopy.

### Ovariectomy

The animals were anesthetized (Ketamine 80 mg/kg + Xylazine 12 mg/kg), both lateral abdominal walls were trichotomized and an incision was made. The ovaries were located, the oviduct was sectioned to remove the ovaries and the incisions were stitched^3,4^. The animals received one dose of antibiotics (Pentabiotic, 24,000 UI/kg) immediately after surgery, and two doses of analgesic (Flunixin meglumine, 2.5 mg/kg), immediately and 24 h after surgery. Animals had two weeks to recover from surgery before the maximal aerobic capacity test. All rats recovered successfully.

### Estradiol (E2) replacement therapy

Immediately after ovariectomy, all animals received a subcutaneously implanted Silastic® capsule containing 360 μg of 17β-estradiol/mL in corn oil or vehicle (corn oil). The Silastic® capsules were made of 20-mm segments of Silastic® tubing (inner/outer diameter: 1.02/2.16 mm). An incision was made in the rat dorsal region (10 mm) to implant the Silastic® capsule using forceps^15^. The incision was subsequently stitched. Silastic® capsules were re-implanted after five weeks in order to maintain concentrations within the physiological range^15,17^. The efficacy of this hormone replacement protocol was confirmed by previous studies^4,15,18,19^.

### Maximal aerobic exercise test

All animals were familiarized with running on the treadmill (0.3 km/h, 10 min/day, 5 days) (Panlab, Havard Apparatus, Spain)^20,21^. All rats were familiarized successfully.

The maximal effort exercise test consisted of 3 m/min increments every 3 min until the rat could no longer keep pace^20,21^. The purpose was to evaluate the maximal aerobic capacity and determine exercise training intensity. Maximal oxygen consumption was accessed during the maximal exercise test (VO_2max_) by indirect calorimetry (Panlab, Harvard Apparatus, Spain) coupled to the treadmill (airflow = 1.0 L/min). VO_2max_ was measured continuously by a computerized system (Metabolism, Panlab, Harvard Apparatus, Spain)^20^.

Mechanical efficiency (ME) was calculated by the formula: ME = (workload/energetic cost) × 100^22^. Workload (W; kgm) was calculated as W = body weight (kg) × TTF × treadmill speed (m min^−1^) × sine (treadmill inclination), where TTF is time to fatigue (min).

### Endurance exercise training

The TR-OVX group performed the exercise on a motor treadmill (Insight®, SP, Ribeirão Preto, Brazil) at low-moderate intensity (∼ 50–70% maximal running speed) 60 min/day, 5 days/week for eight weeks (total of 40 sessions), with a gradual increase in speed from 0.7 to 1.2 km/h weekly^20,22^.

### Transmission electron microscopy

Fragments of the soleus muscle were fixed in Karnovsky’s solution (2.5% glutaraldehyde and 2% paraformaldehyde) in 0.1 M cacodylate buffer pH 7.4 overnight at 4 °C. Then, samples were post-fixed in a mixture of 2% (w/v) osmium tetroxide and 1.5% (w/v) potassium ferrocyanide for a minimum of 2 h to enhance the contrast of organelles. Thereafter, samples were washed in distilled water and kept in 2% uranyl acetate (en bloc staining) overnight, serially dehydrated in graded ethanol baths, and embedded in Epon 812. Finally, 50 nm ultrathin sections were stained with Reynolds lead citrate. Transmission electron microscopy (TEM) was performed using a FEI Tecnai G2-12 Spirit at 80 kV. The images were acquired in a SIS-MegaView 3 CCD camera with 1376 × 1070 pixels. Twenty-four electron micrographs per animal were taken at a × 11,000 magnification. Images were randomly selected from central parts of muscle fibers and were analyzed with ImageJ. Volume densities (Vv) of mitochondria were determined with the classic point counting method using a 252-point-grid (500 × 500 nm grid) projected onto each image^23,24^.

### Redox status and antioxidant enzyme activities

The entire soleus muscle samples were defrosted gradually from − 80 to 4 °C. The left soleus muscles were processed for oxidative stress evaluation. Thereafter, they were homogenized in extraction solution (1 mL/muscle 100 g) containing PBS 01x (125 mL), NaCl (2.925 g), BSA (0.625 g), EDTA (46,5 mg), PMSF (2.125 mg), benzethonium chloride (5.6 mg), Tween 20 (62.5 µL), aprotinin (2.5µ) using an manual macerator. To evaluate the level of lipid peroxidation in the skeletal muscle, 1 mL of the homogenate was centrifugated at 5000×*g* for 5 min at 4 °C. For the analysis of the activity and protein expression of the antioxidant enzymes in the skeletal muscle, 1 mL of the homogenate was centrifugated at 10,000×*g* for 5 min at 4 °C. The Bradford method using bovine serum albumin was used as a standard to determine the sample’s protein levels^25^. The thiobarbituric acid reaction with malondialdehyde was used to determine lipid peroxidation by thiobarbituric acid reactive substances (TBARS) levels^26^. The ferric reducing ability of plasma (FRAP), i.e., the reduction of ferric-tripyridyltriazine [Fe(III)-TPTZ] complex to ferrous-tripyridyltriazine [Fe(II)-TPTZ] was used to determine the total antioxidant capacity^27^. The quantification of superoxide dismutase activity (SOD) was based on the inhibition of the reaction between O_2_^**∙**−^ and pyrogallol^28^. Catalase activity (CAT) was determined by measuring the decrease in H_2_O_2_ absorbance at 240 nm^29^.

### Inflammatory biomarkers

The entire soleus muscle samples were defrosted gradually from − 80 to 4 °C. The right soleus muscles were processed for inflammatory analyses. Thereafter, they were homogenized in extraction solution (1 mL/muscle 100 g) containing PBS 01x (125 mL), NaCl (2.925 g), BSA (0.625 g), EDTA (46.5 mg), PMSF (2125 mg), benzethonium chloride (5.6 mg), Tween 20 (62.5 µL), aprotinin (2.5µ) using a manual macerator. The homogenate was then centrifugated at 10,000×*g* for 10 min at 4 °C. The supernatant was separated and used for analyses of IL-6, IL-10, and TNF-α according to the manufacturer’s instructions by ELISA kits (DuoSet, R&D Systems, United States).

### Statistical analyses

Data are reported as mean ± standard error (S.E.M.). Differences between groups were analyzed using one or two-way ANOVA followed by Dunnet posthoc test. The confidence interval (CI) and effect size (ES) for each significant difference is also shown. The correlation between variables was evaluated using the Pearson coefficient. The significance level for all tests was set at 5%. Statistical analyses were performed with GraphPad Prism 5.0 and G *Power* 3.1.9.2.

### Ethics approval

All surgical procedures and protocols used were approved by Animal Use Ethics Committee of the Universidade Federal dos Vales do Jequitinhonha e Mucuri (protocol nº015/2019) and conducted in accordance with National Institute of Health (NIH) Guide for the Care and Use of Laboratory Animals. Of note, we confirm that our work is reported as described by the ARRIVE guidelines.

### Consent for publication

The researchers of this study confirm that they have given due consideration to protecting the intellectual property associated with this work and that there are no impediments to publication, including the timing of publication, with respect to intellectual property. In so doing, we confirm that we have followed the regulations of our institutions concerning intellectual property.

## Results

**Figure 1:**
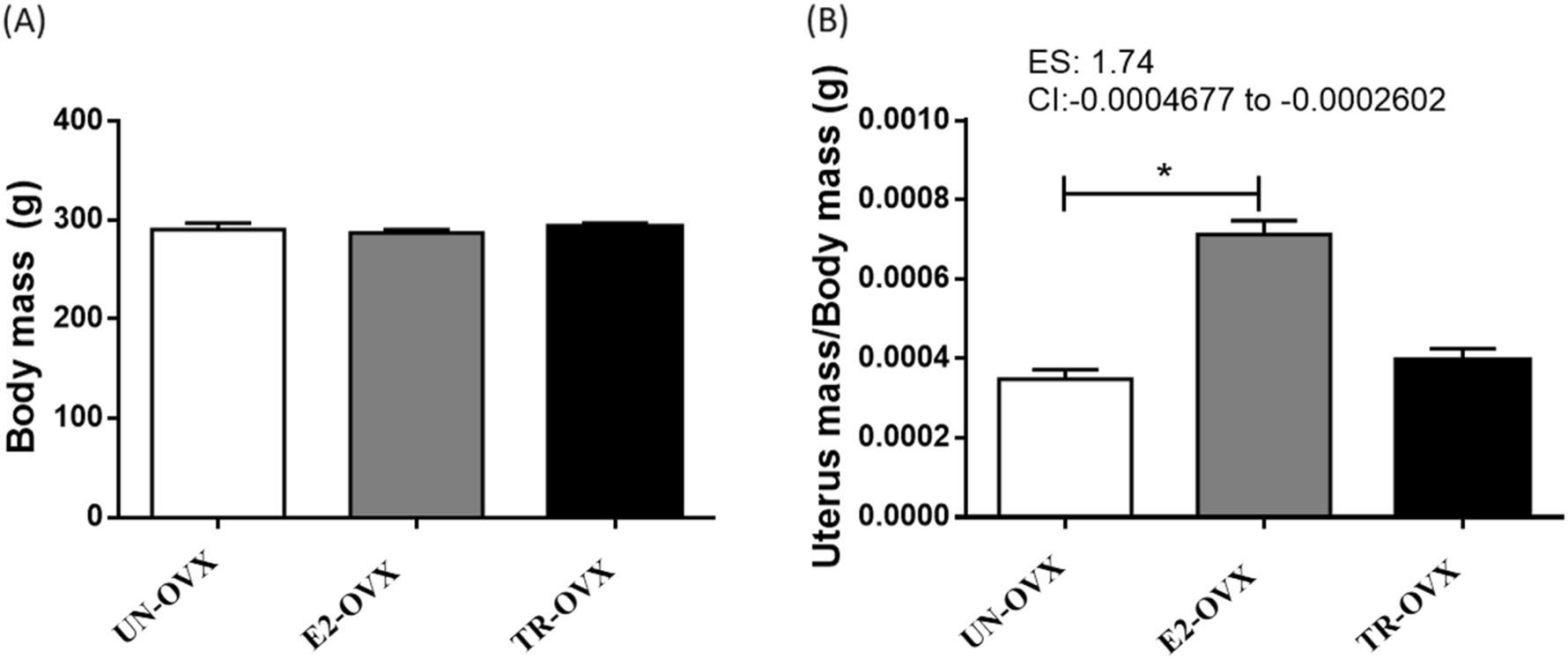
(**A**) Body mass and (**B**) uterus mass/body mass ratio of untrained ovariectomized rats (UN-OVX), ovariectomized rats treated with Estradiol replacement (E2-OVX), and trained ovariectomized rats (TR-OVX). N = 10 per group. Data are presented as mean ± S.E.M. 95% confidence interval (CI). Effect size (ES) *p < 0.05 (TR-OVX vs E2-OVX). One-way ANOVA followed by Dunnet posthoc test.

Figure 1 presents the body mass and the uterus/body mass ratio. Final body mass did not differ between groups after 10 weeks of ovariectomy (UN-OVX: 290.50 ± 6.38 g; E2-OVX: 287.0 ± 3.59 g; TR-OVX: 293.00 ± 3.88 g, Fig. 1A). However, the uterus/body mass ratio was higher in the E2-OVX rats compared with both UN-OVX and TR-OVX groups (E2-OVX: 0.00070 ± 0.00003 g; UN-OVX: 0.00030 ± 0.00002 g; and TR-OVX: 0.00039 ± 0.00002 g, Fig. 1B).

Table 1 shows the results from the maximal aerobic capacity test. Before the training protocol, VO_2max_, mechanical efficiency, final distance and time, and average speed were similar among the groups. After the training period, mechanical efficiency improved only in TR-OVX rats. Moreover, VO_2max_, mechanical efficiency, final distance and time, and average speed were higher in the TR-OVX rats compared with both UN-OVX and E2-OVX rats (Table 1).

**Table 1:** Aerobic capacity.

Measurement	UN-OVX (n = 10)	E2−OVX (n = 10)	TR-OVX (n = 10)	CI (TR vs E2)	CI (UN vs TR)	ES	p^1^	p^2^	p^3^
**VO**_**2max**_** (mL kg**^**-1**^** min**^**−1**^**)**									
Initial	30.34 ± 0.36	30.57 ± 0.45	29.35 ± 0.91	− 2.53 to 3.00	− 3.76 to 1.78	1.73	< 0.001	< 0.001	0.055
Final	25.48 ± 0.75	24.84 ± 0.62	30.59 ± 0.62*	− 3.41 to 2.12	2.32 to 7.87				
**Mechanical efficiency (%)**									
Initial	18.98 ± 0.75	19.55 ± 0.81	21.91 ± 1.20	− 0.05 to 0.06	− 0.02 to 0.08	0.95	< 0.001	< 0.001	< 0.001
Final	22.94 ± 1.94	23.64 ± 1.94	37.51 ± 2.94*^∆^	− 0.04 to 0.06	0.09 to 0.20				
**Distance (m)**									
Initial	233.00 ± 15.44	248.40 ± 22.08	255.20 ± 13.70	− 90.97 to 121.70	− 84.10 to 128.60	1.56	< 0.001	0.079	< 0.001
Final	162.5 ± 21.74	156.00 ± 16.99	530.40 ± 72.85*	− 112.80 to 99.85	262.60 to 475.30				
**Time (s)**									
Initial	1200.00 ± 42.19	1237.00 ± 58.85	1262.00 ± 36.38	− 195.90 to 268.70	− 170.50 to 294.10	1.49	< 0.001	0.635	< 0.001
Final	970.70 ± 71.99	955.20 ± 60.94	1846.75 ± 93.69*	− 247.80 to 216.80	643.40 to 1108.00				
**Speed (m/s)**									
Initial	21.8 ± 0.70	22.5 ± 1.00	22.5 ± 0.50	− 0.20 to 0.27	− 0.20 to 0.27	1.41	< 0.001	0.640	< 0.001
Final	18.0 ± 1.00	17.7 ± 1.00	32.3 ± 1.50*	− 0.25 to 0.22	0.62 to 1.10				

Data are presented as mean ± S.E.M. 95% confidence interval (CI). untrained ovariectomized rats (UN-OVX), ovariectomized rats treated with Estradiol replacement (E2-OVX), and trained ovariectomized rats (TR-OVX). VO_2max_: maximal oxygen consumption. N = 10 per group. Effect size (ES). p1 interaction, p2 time, p3 treatment. *TR-OVX vs UN-OVX and E2-OVX. ^∆^TR-OVX Final vs TR-OVX Initial. Two-way ANOVA followed by Dunnet posthoc test.

**Figure 2:**
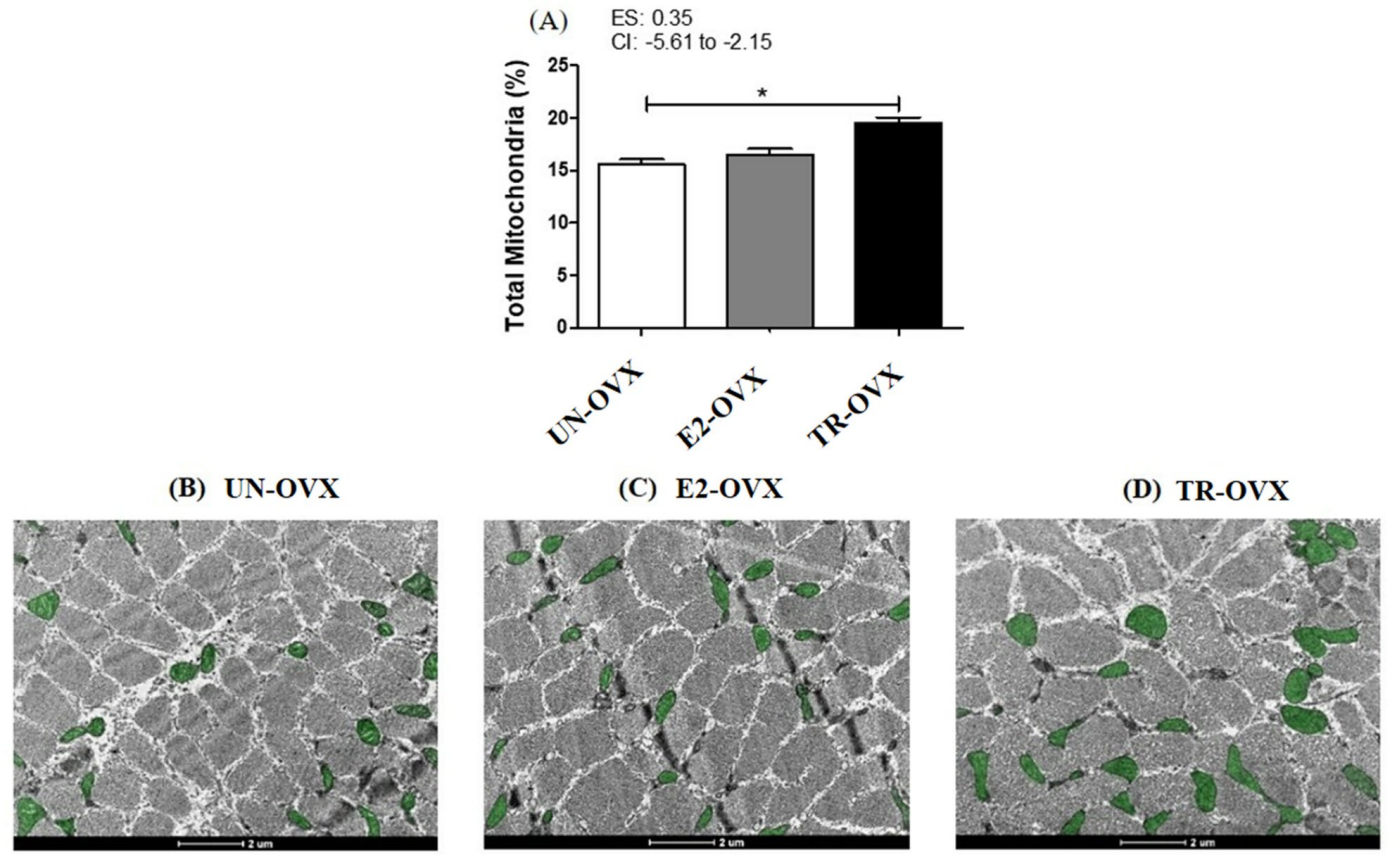
Mitochondrial muscle density. (**A**). Quantification of total mitochondria muscle density in mid-belly fragments of the right soleus from untrained ovariectomized rats (UN-OVX), ovariectomized rats treated with Estradiol replacement (E2-OVX), and trained ovariectomized rats (TR-OVX). N = 72 fields from three animals per group. (**B**–**D**) Transmission electron micrographs (TEM) of transverse sections of muscle fibers (ultra-structural view), in which mitochondria is highlighted in green. Data are reported as mean ± S.E.M. 95% confidence interval (CI). Effect size (ES) *p < 0.01. One-way ANOVA followed by Dunnet posthoc test.

Figure 2 highlights the results of skeletal muscle mitochondrial density. Of note, the skeletal muscle mitochondrial density was higher (~ 20%) in the TR-OVX compared with both E2-OVX and UN-OVX groups (UN-OVX: 15.59 ± 0.47%; E2-OVX: 16.50 ± 0.59%; TR-OVX: 19.48 ± 0.57%; Fig. 2A–D).

**Figure 3:**
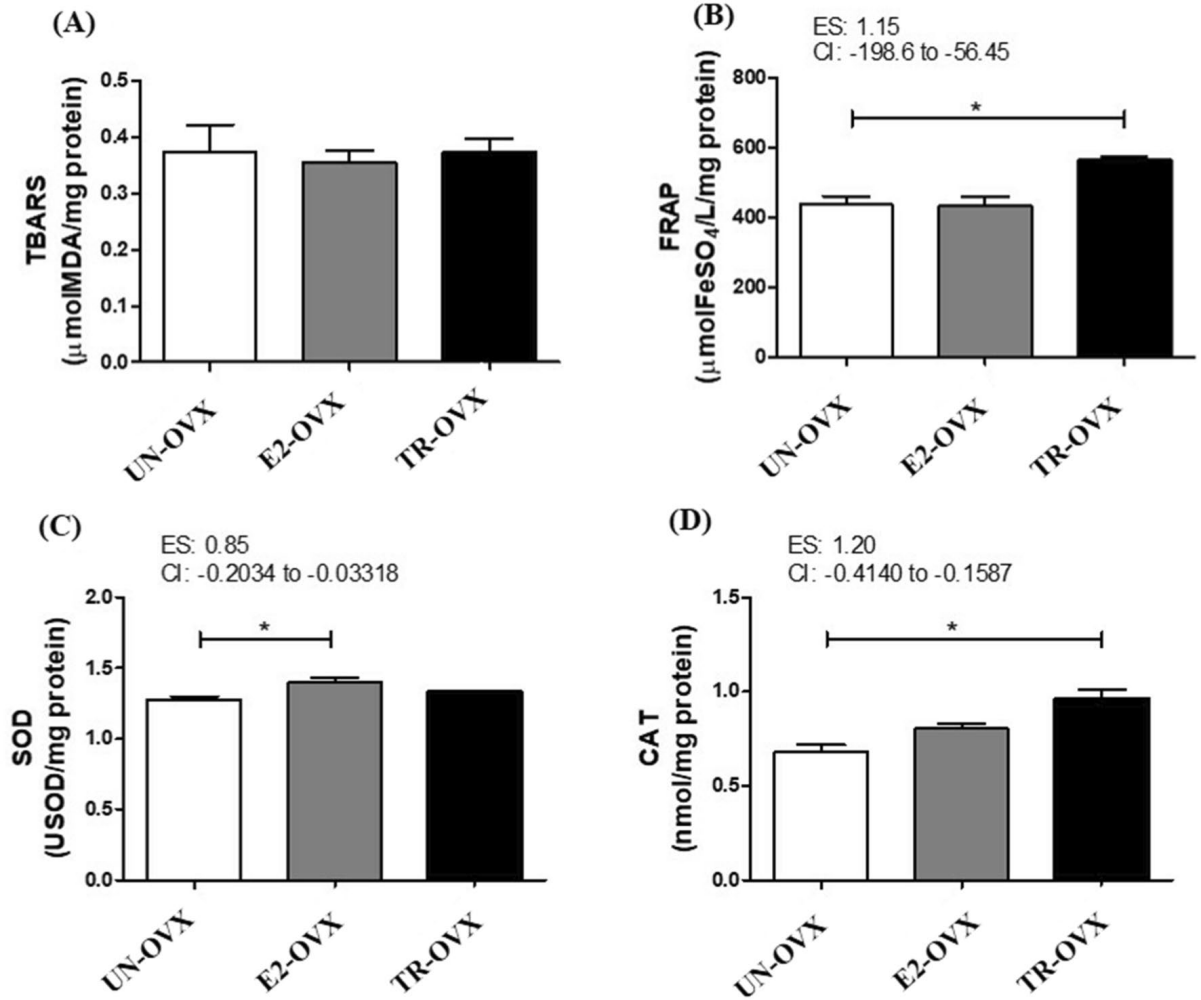
Redox status and antioxidant enzymes assays in the left soleus muscle of untrained ovariectomized rats (UN-OVX), ovariectomized rats treated with Estradiol replacement (E2-OVX), and trained ovariectomized rats (TR-OVX). (**A**). Thiobarbituric acid reactive substances assay (TBARS). (**B**). The ferric reducing ability of plasma (FRAP). (**C**). Superoxide dismutase activity (SOD). (**D**). Catalase activity (CAT). N = 10 per group. Data are reported as mean ± S.E.M. 95% confidence interval (CI). Effect size (ES) *p < 0.01. One-way ANOVA.

Figure 3 shows the results of muscle redox status. TBARS levels did not differ among the groups (UN-OVX: 0.37 ± 0.04; E2-OVX: 0.35 ± 0.01; TR-OVX: 0.37 ± 0.02mmolMDA/mg protein, Fig. 3A). The total antioxidant capacity (FRAP) levels (UN-OVX: 436.10 ± 25.06; E2-OVX: 433.20 ± 23.94; TR-OVX: 563.60 ± 10.80 mmolFeSO4/L/mg protein, Fig. 3B) and catalase activity (UN-OVX: 0.67 ± 0.03; E2-OVX: 0.80 ± 0.026; TR-OVX: 0.96 ± 0.04 nmol/mg protein, Fig. 3C) were higher in the TR-OVX compared with both E2-OVX and UN-OVX groups. Otherwise, SOD activity was higher in the E2-OVX compared with both TR-OVX and UN-OVX rats (UN-OVX: 1.28 ± 0.01; E2-OVX: 1.40 ± 0.03; TR-OVX: 1.33 ± 0.00USOD/mg protein, Fig. 3D).

**Figure 4:**
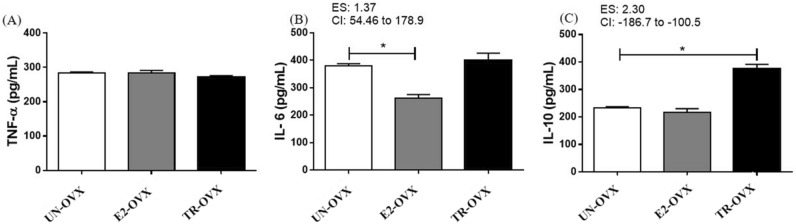
Inflammatory biomarkers assays in the right soleus muscle of untrained ovariectomized rats (UN-OVX), ovariectomized rats treated with Estradiol replacement (E2-OVX), and trained ovariectomized rats (TR-OVX). (**A**). Tumour necrosis factor-alpha (TNF-α). (**B**). Interleukin 6 (IL-6). (**C**). Interleukin 10 (IL-10). N = 10 per group. Data are reported as mean ± S.E.M. 95% confidence interval (CI). Effect size (ES) * p < 0.01. One-way ANOVA.

Figure 4 presents the results of inflammatory biomarkers. TNF-α levels did not differ among the groups (UN-OVX: 283.4 ± 3.343; E2-OVX: 284.4 ± 6.652; TR-OVX: 271.5 ± 4.173 pg/mg, Fig. 4A). However, the IL-6 levels were lower (~ 44%) in the E2-OVX group compared with both TR-OVX and UN-OVX groups (UN-OVX: 378.9 ± 8.875; E2-OVX: 262.3 ± 13.13; TR-OVX: 398.5 ± 27.53 pg/mg, Fig. 4B), and the IL-10 levels were higher (~ 61%) in the TR-OVX rats compared with the E2-OVX and UN-OVX rats (UN-OVX: 232.3 ± 5.249; E2-OVX: 216.0 ± 14.18; TR-OVX: 375.9 ± 15.97 pg/mg, Fig. 4C).

**Figure 5:**
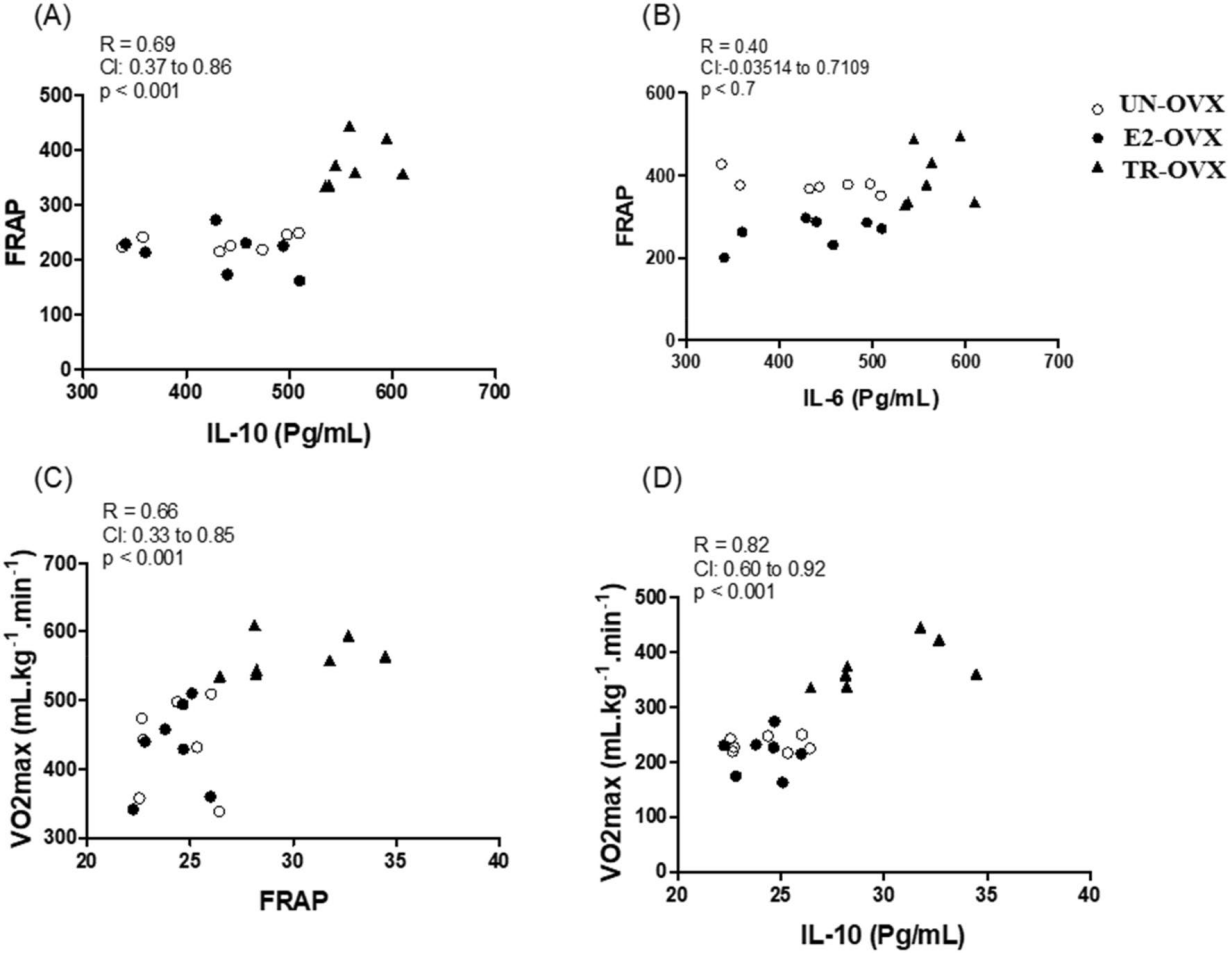
Correlations of the ferric reducing ability of plasma (FRAP) with interleukin 10 (IL-10) (**A**), interleukin 6 (IL-6) (**B**), and maximal oxygen consumption (VO_2max_) with FRAP (**C**) and IL-10 (**D**). The open circle (white) represents untrained ovariectomized rats (UN-OVX). A closed circle (black) represents ovariectomized rats treated with Estradiol replacement (E2-OVX). A closed triangle (black) represents trained ovariectomized rats (TR-OVX).

The correlations of FRAP with IL-10, IL-6, and VO2max with FRAP and IL-10 are shown in Fig. 5. The analyses demonstrated a moderate positive correlation of FRAP with IL-10 (R squared 0.69, p < 0.001, Fig. 5A) while IL-6 showed no significant correlation with FRAP (R squared 0.40, p < 0.001, Fig. 5B). Additionally, we also observed a moderate positive correlation between FRAP and VO2max (squared 0.66, p < 0.001, Fig. 5C), and a strong positive correlation between IL-10 and VO2max (squared 0.82, p < 0.001, Fig. 5D).

## Discussion

The present study was designed to compare the effects of endurance exercise training versus E2 replacement therapy on mitochondrial density, redox status, and inflammatory select biomarkers in the skeletal muscle of OVX rats. The most important finding is that both therapies exert beneficial effects on OVX rat muscles, but endurance exercise therapy was superior to E2 replacement therapy. Endurance exercise training prevented the reduction of aerobic capacity and improved mechanical efficiency. In addition, skeletal muscle mitochondrial density, CAT activity, FRAP, and IL-10 levels were higher in the exercised group. However, in OVX rats, the muscle SOD activity was higher and IL-6 levels lower only in the E2 replacement therapy.

Studies demonstrated that ovariectomy reduces rats’ performance during maximal exercise tests^21,30^. This physical performance deterioration in rats may be a consequence of the decrease in skeletal muscle mitochondrial content and function induced by ovariectomy^4,5^.

In our study, endurance exercise training effectively prevented VO2max reduction, improved mechanical efficiency, and and promoted higher skeletal muscle mitochondrial density (an important physiological indicator of muscle mitochondrial function improvement)^31^ in OVX rats. On the other hand, E2 replacement therapy did not improve any of these parameters. It is noteworthy that ovariectomy may increase mitochondrial ROS production^3–5^, favoring a pro-oxidant and inflammatory status leading to progressive mitochondrial dysfunction and cell death^10,11^. To our knowledge, this is the first study highlighting the effects of endurance exercise training versus E2 replacement therapy on the skeletal muscle mitochondrial density from OVX rats using transmission electron microscopy, the "gold standard" to measure mitochondrial content. This assay exhibits more accuracy in measuring mitochondrial content than other measures, such as transcriptions factors levels, once changing in transcriptions factors levels does not necessarily induce changes in mitochondrial content.

Our results indicate that both E2 replacement therapy and endurance exercise training may protect OVX rat muscle from oxidative stress through distinct pathways. For example, while the SOD activity was higher in the E2 replacement therapy group, the CAT activity was higher in the endurance exercise training group. However, only endurance exercise training had higher total antioxidant capacity (FRAP) compared with the other OVX groups.

Our data also revealed that the skeletal muscle IL-6 levels were lower only in the E2 replacement therapy group compared to the other OVX groups. The complex physiological role of IL-6 has been a matter of debate. While some studies suggest IL-6 as an immune-modulatory cytokine that induces low-grade inflammation in some chronic diseases and a detrimental intramuscular modulatory factor in specific conditions such as cachexia, other studies suggest IL-6 as a key factor that induces positive effects on muscle metabolism and myogenesis^9^. Despite these controversial effects, we believe that the IL-6 reduction induced by E2 replacement therapy in OVX rat skeletal muscle is beneficial and contributes to an anti-inflammatory profile during menopause. Accordingly, a recent study indicated that E2 replacement therapy improved the IL6-induced mitochondrial dysfunction through the activation of both GPER and Erα receptors^32^. However, the mechanism behind the protective effect of E2 on mitochondrial dysfunction induced by a proinflammatory profile in OVX rats remains unknown, and a complete characterization of the modulatory role of E2 replacement therapy on intramuscular cytokines deserves future investigations.

Although endurance exercise training did not change IL-6 muscle levels in OVX rats, the TR-OVX group presented higher levels of IL-10 compared with both UN-OVX and E2-OVX groups. IL-10 is an important anti-inflammatory myokine that down-regulates pro-inflammatory signaling and protects the muscle against oxidative damage^10,11,33^. Moreover, the positive correlation between FRAP, IL-10 and VO2max reinforces the contribution of endurance exercise training to the improvement of total antioxidant capacity and anti-inflammatory status in the OVX rat muscle. Of note, previous studies showed that IL-10 expression is coupled to the nuclear transcriptional network of mitochondrial biogenesis^34^ and the genetic deletion for IL-10 increases damaged mitochondria in skeletal muscles, reinforcing the idea of the benefits of endurance exercise training through mitochondria-cytokine crosstalk^35^.

The probable mechanisms underlying the improvement in skeletal muscle mitochondrial density, redox balance, and anti-inflammatory profile induced by endurance exercise training in OVX rats must be further elucidated. Furthermore, although it is plausible that there would be an additive effect of E2 replacement therapy and endurance exercise training on the evaluated parameters in our study, this experimental design is beyond the scope of the present study and deserves future investigations.

## Conclusion

Overall, endurance exercise training compared to E2 replacement therapy was effective to maintain aerobic capacity and improve mechanical efficiency in skeletal muscle of OVX rats. In addition, endurance exercise training compared to E2 replacement therapy raises the skeletal muscle mitochondrial content and tends to balance the redox and inflammatory status in the skeletal muscle of OVX rats. Thus, endurance exercise training may be an alternative therapy to hormonal replacement for the treatment of musculoskeletal disabilities during menopause.

## Data Availability

The datasets used and/or analyzed during the current study are available from the corresponding author on reasonable request.
